# Effect of venovenous extracorporeal membrane oxygenation on the heart in a healthy piglet model

**DOI:** 10.1186/1749-8090-8-163

**Published:** 2013-06-28

**Authors:** Juanhong Shen, Wenkui Yu, Jialiang Shi, Qiyi Chen, Yimin Hu, Juanjuan Zhang, Tao Gao, Fengchan Xi, Jianfeng Gong, Changsheng He, Ning Li, Jieshou Li

**Affiliations:** 1 Research Institute of General Surgery, Jinling Hospital, Medical School of Nanjing University, 305 East Zhongshan Road, Nanjing 210002, Jiangsu, China

## Abstract

**Background:**

Cardiac function is important for patients treated by venovenous extracorporeal membrane oxygenation (VV ECMO), but data about the effect of VV ECMO on the heart in nonneonates is absent. We studied the effect of VV ECMO on cardiac performance, cardiomyocyte and mitochondria in an animal model.

**Methods:**

Twelve farm piglets were randomly assigned into two groups: control group and ECMO group. In the ECMO group, ECMO cannulaes were placed and ECMO was instituted. Hemodynamics was recorded at baseline, 1 hour after induction, and every 4 hours thereafter, to assess the cardiac performance. All animals were monitored for 24 hours and were euthanized and myocardium was harvested. Myocardial histology, ultrastructure of cardiomyocyte and mitochondria were observed, and activities of mitochondrial complexes I-V were measured, to assess the effect to cardiomyocyte and mitochondria.

**Results:**

Hemodynamics were stable in each group of animals throughout the experiment. Interstitial edema, disorderd and dissolved of focal myofilament, morphological deformations of mitochondria were observed in the ECMO group. The activities of mitochondrial complexes were decreased in the ECMO group, and complex I and IV reached significance.

**Conclusions:**

VV ECMO therapy is associated with changes of ultrastructure and function of cardiomyocyte and mitochondria, inducing myocardium injury. However, the injury was mild and had no effect on the cardiac performance for healthy piglets.

## Background

Venovenous extracorporeal membrane oxygenation (VV ECMO) is a life-saving treatment for severe respiratory failure patient refractory to conventional therapy. VV ECMO maintains sufficient tissue oxygenation and carbon dioxide elimination, so that ventilator settings can be reduced which minimizes ventilator-induced lung injury, and provides time for lung to rest and recovery [1,2]. The improved survival of patients randomized to the ECMO arm in the CESAR research [3] and the successful use of ECMO in the 2009 H1N1 pandemic [4] brought ECMO into the spotlight of the world, and VV ECMO is coming into wider use [5]. However, the overall survival of VV ECMO for severe respiratory failure patients was about 50%-60% [6-8], remains for further improvement.

VV ECMO does not provide direct cardiac support, which depends on intrinsic cardiac function to maintain cardiac output. In the clinical practice, we found that there are some patients developing cardiac dysfunction during VV ECMO therapy, and finally lead to adverse outcome. Therefore, we postulate that myocardial dysfunction plays an important role in determining the outcome of patients treated by VV ECMO, and the possibility of VV ECMO inducing cardiac injury is important.

Very little is known about the effect of VV ECMO on the heart, limited to some clinical observations in neonates [9,10], and absent in pediatric and adult populations. Strleper et al. [9] evaluated the effect of VV ECMO on cardiac performance by echocardiography in 15 infants, and found no deleterious effect. Roberts et al. [10] reported that VV ECMO is safe for inotrope dependent neonates. But it remains unclear whether VV ECMO has some potentially adverse effect on the heart, especially for nonneonatal population.

The aim of the study was to investigate the effect of VV ECMO on the heart in nonneonatal population. Patients with severe respiratory failure have a high prevalence of cardiac dysfunction [11,12]. To avoid the confounding effect of the disease condition, we performed this study in a healthy piglet model. Hemodynamics were monitored to evaluate cardiac performance. As the fundamental of cardiac function, cardiomyocyte and mitochondria were studied to find slighter and earlier stage of changes.

## Methods

This study was approved by the Animal Care Committee of Jinling Hospital. All animals received humane care in accordance with the “Principles of Laboratory Animal Care” formulated by the Ministry of Health of the People’s Republic of China.

### Animal preparation

Twelve farm piglets of either sex (25–35 kg) were fasted for 24 hrs. Anesthesia was induced with ketamine (20 mg/kg), diazepam (8 mg/kg), and atropine (0.1 mg/kg) intramuscularly, maintained with ketamine (10-20 mg/kg/hr) and diazepam (8 mg/kg/hr) intravenously. The animals were intubated through a cervical tracheotomy, and ventilated on volume-control mode with room air at a tidal volume setting of 5–8 mL/kg, positive end expiratory pressure of 5 mmHg, and respiratory rate of 15 breaths per minute. Internal jugular vein and femoral arterial catheters were aseptically placed for intravenous access, blood pressure monitoring, and sample collection, pulmonary artery catheter was inserted in the proximal pulmonary artery via the jugular vein catheter for monitoring of pulmonary artery pressure (PAP) and pulmonary artery occlusion pressure (PAOP).

### Experimental protocol

After instrumentation and baseline data collection, animals were randomly assigned into two groups: control group and ECMO group (n =6/each). In the ECMO group, after a 150 U/kg intravenous bolus of heparin, ECMO cannulaes (15 F, Medtronic, Minneapolis, Minnesota) were placed in the superior vena cava through internal jugular vein and inferior vena cava through femoral vein, and ECMO circulation was instituted. Placement of the cannulaes was confirmed via ultrasonograph. Heparin was infused to keep activated clotting time of 180–220 seconds after insertion.

The ECMO system consisted of a membrane oxygenator and tubing (Quadrox PLS, Maquet, Rastatt, Germany), a centrifugal pump (Rotaflow Console, Maquet, Rastatt, Germany), a heat exchange (Heater-Cooler Unit HCU 30, Maquet, Rastatt, Germany). The circuit was primed with 500 ml hydroxyethyl starch 130/0.4 and 200-300 ml Ringer’s lactate. Blood in circuit was drained from femoral vein cannulae and infused into internal jugular vein cannulae at a flow rate of 50 ml/(kg.min). 100% oxygen was given at a flow rate equal to the blood flow rate.

Hemodynamic parameters, including heart rate (HR), mean arterial pressure (MAP), mean pulmonary artery pressure (MPAP) and pulmonary artery occlusion pressure (PAOP) were monitored (PiCCO2, PULSION, Munich, Germany) and recorded at baseline, 1 hour after induction, and every 4 hours thereafter until 24 hours. All pressures were determined at end-expiration. Each animal received lactated Ringer’s solution at a rate of 3 ml/kg/h, and bolus fluid was provided as required to maintain MAP above 60 mmHg. The whole volume of fluid intake of each animal was recorded. Temperature, Serum pH, glucose and ionized calcium concentration were monitored and maintained normally.

All animals were monitored for 24 hours and were euthanized with a bolus injection of potassium chloride (40 ml, 0.1 g/ml). Myocardium was harvested immediately for further measuring.

### Myocardial histology examination

Samples from the apex of left ventricular muscle were fixed and dehydrated with 10% formalin, embedded in paraffin, cutted into sections, stained with hematoxylin and eosin (H and E staining), and observed by an optical microscopy (CX41, Olympus, Tokyo, Japan).

### Myocardial ultrastructure examination

Fresh myocardial tissues from the apex of left ventricular muscle were cut into pieces (1*1*1 mm), fixed with 3% glutaraldehyde, flushed with phosphate-buffered saline, fixed with 1% perosmic acid, and dehydrated with acetone. Ultrathin sections were placed on 200-mesh copper grids and double stained with 4% uranyl acetate and 0.2% lead citrate. Sections were examined under transmission electron microscopy (TEM, JEM-1010, JEOL, Tokyo, Japan). Ultrastructure of cardiomyocyte and mitochondria was observed.

The histology and ultrastructure were examined by an independent pathologist blinded to the grouping.

### Mitochondrial isolation and measurement of mitochondrial complexes activities

Heart tissues from the apex of left ventricular muscle were put in the ice-cold homogenization buffer. The mitochondrial pellet was isolated by differential centrifugation [13], and finally resuspended in phosphate buffer. Mitochondrial protein concentration was determined using the method of Bradford [14].

Activities of mitochondrial respiratory chain complexes I-V were measured using spectrophotometry [15]. The activity of complex I was expressed as μmoles of nicotinamide adenine dinucleotide reduced (NADH) oxidized/min/mg protein, the activity of complex II was expressed as μmoles of 2,6-diclorophenol indophenol (DCIP) oxidized/min/mg protein, the activity of complex III was expressed as μmoles of decylubiquinol oxidized/min/mg protein, the activity of complex IV was expressed as μmoles of cytochrome c oxidized/min/mg protein, the activity of complex V was expressed as μmoles of NADH oxidized/min/mg protein.

### Statistical analysis

All the statistical analyses of the data were performed by the SPSS 17.0 software (SPSS Inc., Chicago, Illinois). Data are expressed as mean ± SD or median (interquartile range). Data were statistically analyzed using one-way analysis of variance (ANOVA). Significant results were *post hoc* analyzed, using the least significance difference (LSD) tests. Statistical significance was established as *p* <0 .05.

## Results

There was no difference in body weight and other characteristic between groups.

### Hemodynamics and fluid intake

**Figure 1:**
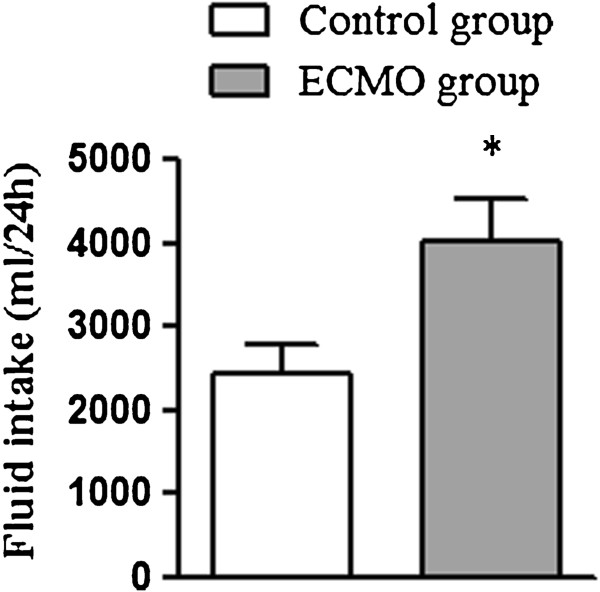
**Volume of fluid intake of each group.** Data are shown as mean ± SD. *P <0.05 vs. control group.

Hemodynamic parameters, including heart rate (HR), mean arterial pressure (MAP), mean pulmonary artery pressure (MPAP) and pulmonary artery occlusion pressure (PAOP) were stable in each group of animals throughout the experiment, and no significance was presented between groups (Table 1). However, fluid intake in the ECMO group was significantly more than the control group (Figure 1).

**Table 1:** Time course of hemodynamics in the two groups

		**Baseline**	**1 h**	**5 h**	**9 h**	**13 h**	**17 h**	**21 h**
HR (beats/min)								
Control group		136 ± 10.1	130 ± 13.7	126 ± 10.3	123 ± 11.8	124 ± 14.5	128 ± 14.5	131 ± 10.4
ECMO group		134 ± 4.9	140 ± 12.4	130 ± 12.5	133 ± 10.4	128 ± 10.9	124 ± 15.2	128 ± 10.4
MAP (mmHg)								
Control group		70 ± 6.5	72 ± 8.5	76 ± 9.3	75 ± 8.5	70 ± 9.1	75 ± 9.9	75 ± 9.3
ECMO group		67 ± 5.0	68 ± 9.5	73 ± 9.4	71 ± 9.1	77 ± 10.6	76 ± 8.5	73 ± 9.9
MPAP (mmHg)								
Control group		17.7 ± 4.0	19.2 ± 4.0	18.2 ± 3.3	19.0 ± 3.6	18.8 ± 3.9	16.7 ± 4.0	18.3 ± 4.0
ECMO group		18.5 ± 3.1	20.2 ± 4.4	19.5 ± 4.5	20.2 ± 4.6	19.3 ± 4.0	19.0 ± 3.1	18.7 ± 3.6
PAOP (mmHg)								
Control group		12.0 ± 2.1	11.7 ± 1.2	11.3 ± 1.9	11.0 ± 0.9	11.2 ± 1.2	11.7 ± 1.9	11.5 ± 1.2
ECMO group		11.2 ± 1.5	11.0 ± 1.1	11.8 ± 1.2	12.0 ± 0.9	12.0 ± 1.7	11.8 ± 1.6	12.0 ± 1.4

Data are shown as mean ± SD. No significance was presented. *HR* heart rate, *MAP* mean arterial pressure, *MPAP* mean pulmonary artery pressure, *PAOP* pulmonary artery occlusion pressure.

### Myocardial histology and ultrastructure changes

**Figure 2:**
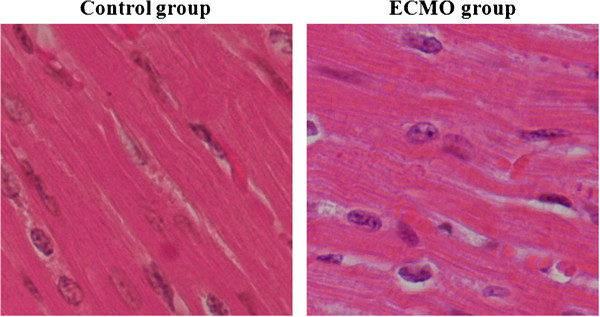
Histology of myocardial with H and E staining under optical microscopy (magnification*400). Myocardial structure was normal in either group, but interstitial edema was observed in the ECMO group.

H and E staining in the control group showed that cardiomyocytes were arranged in rows, the fiber structure of cardiomyocyte was clear. There was no obvious damage to myocardial fibers in the ECMO group, but interstitial edema was observed, myocardial fiber spacing was increased (Figure 2).

**Figure 3:**
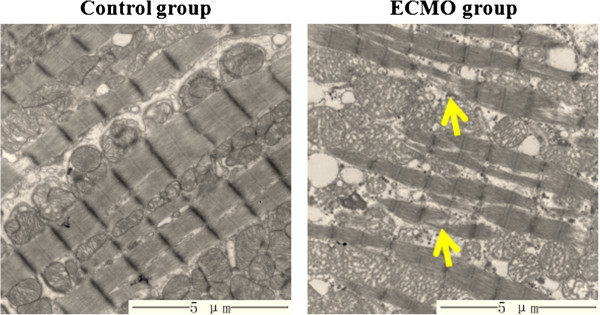
Ultrastructure of cardiomyocyte under TEM (magnification*8000). Ultrastructure of cardiomyocyte was normal in the control group, and mildly disorderd of myofilament and dissolved of focal myofilament (pointed by arrow) were observed in the ECMO group.

Under TEM, samples from the control group showed myofilament was ordered, the structure was clear, the sarcolemma was intact. Samples from the ECMO group showed myofilament was mildly disorderd, dissolved of focal myofilament can be observed (Figure 3).

### Mitochondrial structure and function

**Figure 4:**
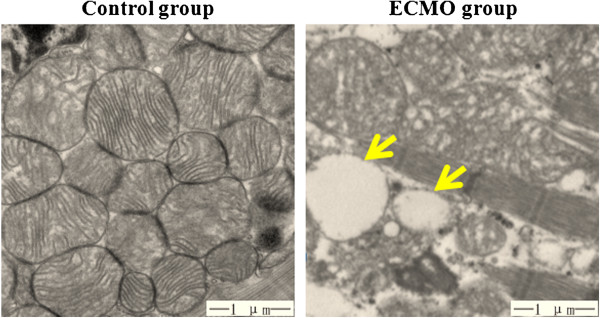
Ultrastructure of mitochondria under TEM (magnification*20000). The mitochondria was normal in the control group; morphological deformations characterized by mitochondrial swollen, decreased density of inner membrane cristae, along with vacuolation(pointed by arrow) were observed in the ECMO group.

The mitochondria from the control group exhibited well-defined double membranes with normal cristae arrangement Abnormal mitochondria can be observed in the ECMO, characterized by mitochondrial swollen, decreased density of inner membrane cristae, along with vacuolation, but the outer membrane was integrated (Figure 4).

**Figure 5:**
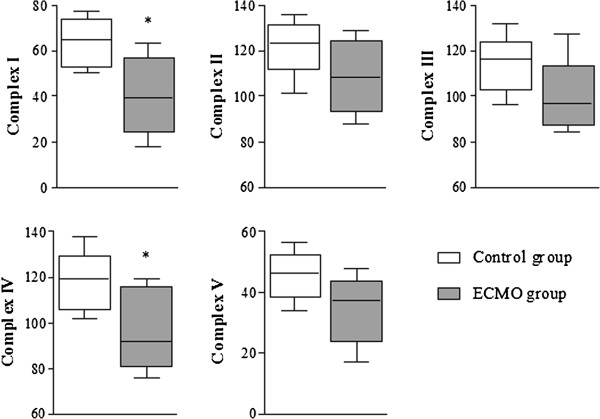
**Enzymatic activities of mitochondrial complex I-V in left ventricular myocardium in each groups of animals.** Box plots indicate median, interquartile range, 10th and 90th percentile. *P <0.05 vs. control group. Units are expressed as: complex I-μmoles of NADH oxidized/min/mg protein, complex II-μmoles of DCIP oxidized/min/mg protein, complex III-μmoles of decylubiquinol oxidized/min/mg protein, complex IV- μmoles of cytochrome c oxidized/min/mg protein, complex V -μmoles of NADH oxidized/min/mg protein.

The activities of mitochondrial complexes were decreased in the ECMO group versus the control group (Figure 5), and activities of complex I and IV reached significance.

## Discussion

As a lifesaving treatment, VV ECMO is becoming more and more essential for children and adults with refractory respiratory failure, and such patients have a high prevalence of cardiac dysfunction [11,12]. VV ECMO does not provide direct cardiac support, which depends on intrinsic cardiac function to maintain cardiac output. Therefore, the condition of cardiac function during VV ECMO is closely associated with the outcome. We present the first detailed laboratory investigation in the effect of VV ECMO on the heart in nonneonatal population.

In this study, the volume of fluid intake was significantly more in the ECMO group. Golej et al. [16] found MAP decreased at the initiation of VV ECMO and increased in a short time. In consideration of the numerous influence factors, we haven`t analyzed the immediate hemodynamic response. But we have observed similar MAP fluctuation at the initiation of VV ECMO and restored quickly after fluid bolus, which contributed a lot to the amount of the fluid taken in the ECMO group, and may associated with myocardium interstitial edema.

In addition, the ECMO group disorderd and dissolved of focal myofilament, morphological deformations of mitochondria and decreased activities of mitochondrial complexes compared with the control group, suggesting that VV ECMO therapy is associated with myocardium injury.

The mechanisms of these findings require further research. Several factors may contribute to it, such as activation of systemic inflammatory response, increase of reactice oxygen species (ROS), and release of toxic substances by ECMO.

The instigation of a systemic inflammatory state with exposure to ECMO is well accepted [17-21]. Fortenberry et al. [19] found that neutrophil was activated and concentration of circulating interleukin (IL)-8 increased significantly after ECMO initiation. Adrian et al. [20] perfused blood in vitro ECMO circuit performed for 24 hours, and found cytokines, including IL-1β, IL-1 receptor antagonist, IL-8, IL-6, tumor necrosis factor α (TNF-α) were increased. Mu et al. [21] found concentrations of circulating IL-6 and IL-10 increased significantly in a hemorrhage-reperfusion piglet model on modern ECMO. The inflammatory cytokines have been implicated in mediating myocardial injury [22-24]. Deng et al. [25] and Hennein et al. [26] found that preoperative left ventricular dysfunction is associated with the degree of proinflammatory cytokine release. Liakopoulos et al. [27] found that cardiac dysfunction occurred after cardiopulmonary bypass was associated with the increase of systemic and myocardial TNF-α, and anti-inflammatory pretreatment with methylprednisolone abolished the increase of TNF-α attenuated myocardial dysfunction.

It is well established that cardiopulmonary bypass is associated with increased production of reactice oxygen species (ROS) [28,29], and similar results were verified during ECMO therapy. Hirthler et al. [17] and Underwood et al. [18] detected that systemic free radical was increased during ECMO. Moller et al. [30] found free oxygen radical scavenging enzymes such as superoxide dismutase and glutathione reductase were decreased during ECMO in healthy lambs, resulted in increased lipoperoxide level. ROS are central mediators of cardiac injury, especially for mitochondria, and finally resulting in cardiac dysfunction [31]. Precious researches indicate that lacking of ROS scavenging enzyme aggravate myocardial injury [32], and antioxidant potentiate cardiac protection [33].

In addition, it is not clear whether the ECMO circuit adds some toxic substances such as endotoxin [17] and plasticizers [34] impact the myocardium.

Despite changes of ultrastructure and function of cardiomyocyte and mitochondria induced by VV ECMO, we found hemodynamics during VV ECMO therapy was stable, suggesting that the injury was mild, and had no effect on the cardiac performance for healthy piglets, consistently with the results of Strieper [9] and Roberts [10].

However, energy is the base to maintain normal cardiac pump function, continuously produced by mitochondrial respiration [35]. Therefore, any alteration of mitochondrial structure and function are fundamental for cardiac function. The changes of mitochondria in this study do not impact the cardiac performance, but may impact the cardiac reserved function, which may essential for a marginally functional heart or when cardiac demand increased under disease conditions.

Furthermore, under disease conditions with severe hypoxia, hypoxia itself plays an important role in myocardial injury [12], and the advantage of VV ECMO in providing adequate oxygenation to the myocardium may be of protective effect. Shen et al. [36] found cardiac dysfunction due to hypoxia coronary perfusion during venoarterial ECMO in a hypoxemic swine model, postulating that VV ECMO may be more adequate for hypoxemic condition. However, abrupt hyperoxia for a hypoxemic heart may induce reoxygenation injury, causing further cardiac injury. Allen et al. [37] and Trittenwein et al. [38] found increased amounts of oxygen free radicals after reoxygenation on cardiopulmonary bypass and ECMO respectively.

Therefore, the effect of VV ECMO on the myocardium and cardiac performance would be more complicated for patients under hypoxemic condition, and should be further investigated. However, we present the potentially adverse effect of VV ECMO alone on the heart for the first time, calling for attention to the impact on heart during VV ECMO, and provide a potentially aspect to improve the survival of VV ECMO.

## Conclusions

In conclusion, the results of our study showed that VV ECMO is associated with changes of ultrastructure and function of cardiomyocyte and mitochondria, inducing myocardium injury. The underlying mechanisms should be further investigated. However, the injury was mild and had no effect on the cardiac performance for healthy piglets.

## Abbreviations

VV: Venovenous; ECMO: Extracorporeal membrane oxygenation; HR: Heart rate; MAP: Mean arterial pressure; MPAP: Mean pulmonary artery pressure; PAOP: Pulmonary artery occlusion pressure; H and E staining: Stained with hematoxylin and eosin; TEM: Transmission electron microscopy; NADH: Nicotinamide adenine dinucleotide reduced; DCIP: 2,6-Diclorophenol indophenol; ROS: Reactice oxygen species; IL: Interleukin; TNF-α: Tumor necrosis factor α.

## Competing interests

The authors declare that they have no competing interests.

## Authors’ contributions

JL, WY, NL, JS contributed to the experiment conception and design; JS, QC, JS, YH, CH participated in the animal experiment and were responsible for histology and ultrastructure section; JZ, TG, FX, JG participated in the animal experiment and were responsible for the biochemistry section; JS did the statistics and drafted the manuscript; WY, NL and JL provided critical revision of the manuscript; all the authors read and approved the final manuscript.
